# Biosynthesis of the Nematode Attractant 2-Heptanone and Its Co-evolution Between the Pathogenic Bacterium *Bacillus nematocida* and Non-pathogenic Bacterium *Bacillus subtilis*

**DOI:** 10.3389/fmicb.2019.01489

**Published:** 2019-07-02

**Authors:** Man Zhu, Xiao’e Xu, Yuhong Li, Pengfei Wang, Shanzhuang Niu, Keqin Zhang, Xiaowei Huang

**Affiliations:** State Key Laboratory for Conservation and Utilization of Bio-Resources, Key Laboratory for Microbial Resources, Ministry of Education, Yunnan University, Kunming, China

**Keywords:** methylketone biosynthesis, 2-heptanone, pathogenic bacterium, interaction between pathogen and host, co-evolution, *Bacillus* sp.

## Abstract

Methylketones are broadly distributed in nature and perform a variety of functions. Most microorganisms are thought to produce methylketone by abortive β-oxidation of fatty acid catalytic metabolism. However, two methylketone synthetase genes in wild tomatoes are reported to synthesize methylketone using intermediates of the fatty acids biosynthetic pathway. In our previous study on Trojan horse-like interactions between the bacterium *Bacillus nematocida* B16 and its host worm, the chemical 2-heptanone was found to be an important attractant for the hosts. So here we used this model to investigate the genes involved in synthesizing 2-heptanone in microorganisms. We identified a novel methylketone synthase gene *yneP* in *B. nematocida* B16 and found enhancement of *de novo* fatty acid synthesis during 2-heptanone production. Interestingly, a homolog of *yneP’* existed in the non-pathogenic species *Bacillus subtilis* 168, a close relative of *B. nematocida* B16 that was unable to lure worms, but GC-MS assay showed no 2-heptanone production. However, overexpression of *yneP’* from *B. subtilis* in both heterologous and homologous systems demonstrated that it was not a pseudogene. The transcriptional analysis between those two genes had few differences under the same conditions. It was further shown that the failure to detect 2-heptanone in *B. subtilis* 168 was at least partly due to its conversion into 6-methyl-2-heptanone by methylation. Our study revealed methylketone biosynthesis of *Bacillus* species, and provided a co-evolution paradigm of second metabolites during the interactions between pathogenic/non-pathogenic bacteria and host.

## Introduction

Methylketones are broadly distributed in natural environments and can be produced by a variety of bacteria, fungi, plants, insects, and even mammals. In bacteria, methylketones, such as 2-heptanone, 2-butanone, 2-petanone, and 3-methyl-1-butanone, are detected in the volatile organic compounds (VOCs) of *Lactobacillus casei* (Gallegos et al., 2017). *Staphylococcus carnosus* produces 2-pentanone and a highly autolytic strain of *Lactobacillus helveticus* is reported to enhance the levels of methylketones during cheese ripening (Fadda et al., 2002; Hannon et al., 2006). In fungi, both 2-heptanone and 2-petanone are detected from a range of species, from unicellular yeast to filamentous fungi (Sunesson et al., 1996). In plants, short-chain methylketones (C_5_–C_11_) are aromatic substances, commonly enriched in essential oil from leaves and fruits; while medium-chain methylketones (C_7_–C_15_) provide resistance to insects (Schwab et al., 2008; Fischer et al., 2013). In animals, methylketones are used as the pheromones or allomone. For example, 2-heptanone functions as honey bee’s alarm pheromone to guide other bees to attack enemies (Torto et al., 2007). Some small arthropods were reportedly paralyzed by 2-heptanone after bitten by honeybees (Papachristoforou et al., 2012). A higher concentration of 2-heptanone in the urine of male *Rattus norvegicus* was found to be more attractive to the females (Zhang et al., 2008).

Despite their extensive distributions and diverse roles, relatively little is known about the biosynthesis of methylketones. In bacteria, it was reported that the production of odd-numbered methylketones via decarboxylation of even-numbered fatty acids (Yu et al., 2000; Dickschat et al., 2005; Maddula et al., 2009). Studies in fungi found fatty acids were oxidized into β-ketoacids followed by β-keto-acyl-decarboxylase to produce methylketones (Kinsella and Hwang, 1976; Qian et al., 2002). But the spores of *Penicillum roqueforti* catalyzed the production of 2-undecanone, 2-heptanone, and 2-pentanone from short-chain alkyl esters of lauric acid, octanoic, hexanoic in an aqueous-organic two-phase system, respectively (Park et al., 2000). Acetone is the common methylketone found in mammals and is produced by oxidation of butyric acid (Kalapos, 1999). Collectively, it is widely believed that odd-numbered methylketones are synthesized from their corresponding even-numbered fatty acids via abortive β-oxidation (Schulz and Dickschat, 2007). However, it was recently shown that in wild tomatoes (*Solanum habrochaites*) that could produce methylketones, the gene expressional levels involved in fatty acid synthesis were much higher than those of fatty acid beta oxidation pathway (Fridman et al., 2005; Yu and Pichersky, 2014). Furthermore, two methylketone synthetase genes *shMKSI* and *shMKSII* have been identified to catalyze the substrate β-ketoacyl-ACP (β-ketoacyl-acyl carrier protein), which was one of the intermediate products in fatty acid synthesis, into the products of methylketone (Fridman et al., 2005). But whether the methylketone synthetase genes exist in the other organisms, and if they do, whether they work similarly as in wild tomatoes remains to be elucidated.

In our previous study about the interactions between the bacterial pathogen *Bacillus nematocida* B16 strain and its host nematode, a Trojan horse-like strategy was demonstrated necessary for the bacterial pathogenesis, in which the VOCs, including 2-heptanone, were secreted as the attractants to lure worms (Niu et al., 2010). In this study, we used this bacterium *B. nematocida* B16 to investigate the methylketone biosynthesis. A novel methylketone synthase gene *yneP* in *B. nematocida* B16 was identified and found to be involved in the production of 2-heptanone. In addition, we compared *B. nematocida* B16 with a close relative *B. subtilis* 168 and found that the latter bacterium also had a similar methyl ketone synthase but that it lacked both 2-heptanone and attractive ability for nematodes. We further traced the potential reasons responsible for the differences of 2-heptanone production between them.

## Materials and Methods

### Bacterial Strains, Plasmids, Primers, and Grow Conditions

The strains and plasmids used in this study are listed in Table 1. The primers used in this study are listed in Table 2. *Escherichia coli* DH5α was used as the host strain for the construction of plasmids. *E. coli* BL21 was used to express the target proteins. *C. elegans* was grown on NGM medium (50 mM NaCl, 20 g/L of agar, 2.5 g/L of peptone, 1.0 mM cholesterol, 1.0 mM CaCl_2_, 1.0 mM MgSO_4_, and 25 mM potassium phosphate at pH 6.0) seeded with *E. coli* OP50. Then the well-fed adult *C. elegans* were prepared by washing three times with M9 buffer and once with water for the following experiments.

**Table 1:** Strains and plasmids used in this study.

Strain or plasmid	Genotype/description	Source or references
**Strains**		
*B. nematocida* B16	Wild Type	Laboratory stock
*B. subtilis* 168	Wild Type	Laboratory stock
*E. coli* DH5α	Clone strain	TaKaRa
*E. coli* BL21	*E. coli* strain for heterologously expressing protein	Laboratory stock
*E. coli* E*yneP*	BL21 heterologously expressing *yneP* of *B. nematocida*	This study
*E. coli* E*ytpA*	BL21 heterologously expressing *ytpA* of *B. nematocida*	This study
*E. coli* E*yneP*’	BL21 heterologously expressing *yneP* of *B. subtilis*	This study
*B. subtilis* E*yneP’*	*B.subtilis* 168 homologously expression *yneP* of *B. subtilis*	This study
BSP1	*B.subtilis* 168 amyE::P*yneP*-627bp-lacZ(Cm^r^)	This study
BSP2	*B.subtilis* 168 amyE::P*yneP*-518bp-lacZ(Cm^r^)	This study
BSP3	*B.subtilis* 168 amyE::P*yneP*-383bp-lacZ(Cm^r^)	This study
BSP4	*B.subtilis* 168 amyE::P*yneP*-356bp-lacZ(Cm^r^)	This study
BSP5	*B.subtilis* 168 amyE::P*yneP*-217bp-lacZ(Cm^r^)	This study
BSP6	*B.subtilis* 168 amyE::P*yneP*-198bp-lacZ(Cm^r^)	This study
BNP1	*B.nematocida* B16 amyE::P*yneP*-628bp-lacZ(Cm^r^)	This study
BNP2	*B.nematocida* B16 amyE::P*yneP*-491bp-lacZ(Cm^r^)	This study
BNP3	*B.nematocida* B16 amyE::P*yneP*-444bp-lacZ(Cm^r^)	This study
BNP4	*B.nematocida* B16 amyE::P*yneP*-229bp-lacZ(Cm^r^)	This study
BNP5	*B.nematocida* B16 amyE::P*yneP*-205bp-lacZ(Cm^r^)	This study
BNP6	*B.nematocida* B16 amyE::P*yneP*-200bp-lacZ(Cm^r^)	This study
**Plasmids**		
pMD-19T	Cloning vector in *E. coli*	TaKaRa
pMD-18T	Cloning vector in *E. coli*	TaKaRa
pET-28a	Expressional vector in *E. coli* BL21	Laboratory stock
pET28a-*yne*P	Recombinant expressional plasmid for expressing *yneP* of *B. nematocida*	This study
pET28a-*ytp*A	Recombinant expressional plasmid for expressing *ytpA* of *B. nematocida*	This study
pET28a-*yne*P’	Recombinant expressional plasmid for expressing *yneP’* of *B. subtilis*	This study
pIs284	Insertion vector to amyE, chloramphenicol resistance, lacZ	From Mitsuo Ogura
pDG148	Expressional vector in *B. subtilis*	From Francois Denizot
pDG148-*yneP’*	Expressional plasmid for homologously expressing *yneP* in *B. subtilis*	This study
pCP115	Gene knockout plasmid in *Bacillus* sp.	Bacillus Genetic Stock Center
pCP115-*yne*P	*yne*P gene knockout plasmid in *B. nematocida*	This study

**Table 2:** The primers used in this study.

Primer	Nucleotide sequence (5′→3′)	Functions
*FytpA*	GGATCCATGTGGACTTGGAAAGCAG	pET28a-*ytp*A
R*ytpA*	CTCGAGTCAAATATACTGATCTGTAAAG	pET28a-*ytp*A
F*yneP*	GGATCCTTGCATGTGTCAAAAAAAG	pET28a-*yne*P
R*yneP*	CTCGAGTTATTTTTTTGCCTTTTCGT	pET28a-*yne*P
F*yneP’*	GGATCCTTGCATGTGTCAAAAAAAG	pET28a-*yne*P’
R*yneP’*	CTCGAGCTATTTTTTGGCCTTTTCAT	pET28a-*yne*P’
F*bsyneP’*	GTCGACTTGCATGTATCAAAAAAA	pDG148-*yneP’*
R*bsyneP’*	GCATGCCTAGTGGTGATGGTGATGATGTTTTTTGGCCTTTTCATAT	pDG148-*yneP’*
BSP1	GAATTCCATTATCACCTCAATCAT	Reporter gene upstream 1
BSP2	GAATTCATAAAAACATTGATATTTAC	Reporter gene upstream 2
BSP3	GAATTCAACTGCAGCAAAAGAC	Reporter gene upstream 3
BSP4	GAATTCGAGAAAAACGTTTACTAC	Reporter gene upstream 4
BSP5	GAATTCGGCTTCGTTATCAAAAA	Reporter gene upstream5
BSP6	GAATTCAACACGTGGAAAATTTG	Reporter gene upstream 6
BSPRV	GGATTCTAACGAGATCAACAGGAA	Reporter gene downstream
BNP1	GAATTCTCACATGATTGGCTTTCC	Reporter gene upstream 1
BNP2	GAATTCTAAAAACATTGATATTTACTTATG	Reporter gene upstream 2
BNP3	GAATTCTGTTTTGCGTCAGGTAGA	Reporter gene upstream 3
BNP4	GAATTCTTGTGAGAAAATTGTGATGG	Reporter gene upstream 4
BNP5	GAATTCTTCTATTAAGGTTCTTTTGG	Reporter gene upstream 5
BNPRV	GGATTCTTTATCAGGGTCTCCGCCGA	Reporter gene downstream

*The underlined sequences mean the restriction enzyme cutting sites.*

### Plasmids Construction, Subcloning, and Transformation

To confirm the functions of candidate genes in methylketone synthesis, the full opening reading frames (ORF) of target genes were amplified via PCR using the primers of Exp F*yneP* and R*yneP* for the *yneP* gene, F*ytpA* and R*ytpA* for the *ytpA* gene in *B. nematocida* B16, and F*yneP’* and R*yneP*’ for the *yneP*’ gene in *B. subtilis* 168. Those DNA fragments obtained from gel recovery were ligated to the pMD-19T vector respectively, and then the recombinant plasmid was transformed into *E. coli* DH5α competent cells. After the positive transformants were validated by PCR reactions and DNA sequencing, they were digested with *Bam*HI and *Xoh*I at the primer-incorporated restriction sites, and then inserted into a *Bam*HI/*Xoh*I digested pET28a vector to obtain the recombinant plasmids pET28a-*yne*P, pET28a-*ytp*A, and pET28a-*yneP’*. Those three recombinant plasmids were finally transformed into the competent cells of *E. coli* BL21 again to express the target proteins.

In order to over-express *yneP’* in its native host *B. subtilis* 168, the *yneP’* ORF from *B. subtilis* 168 was amplified using primers F*bsyneP’* and R*bsyneP’* that had the primer-incorporated restriction sites of *Sal*I and *Sph*I. The vector pDG148 was used to construct the recombinant plasmid pDG148-*yneP’* and this recombinant plasmid was finally transformed into competent cells of *B. subtilis* 168.

### Expression of Methylketone Synthase

Selected positive transformants were cultured by incubation overnight at 37°C with shaking in 5 ml of LB supplemented with 50 μg/ml kanamycin. After a 3 ml overnight culture was transferred into 300 ml of fresh LB medium and grown until an OD600 reached 0.3, 0.1 mM IPTG was then added to induce the protein expression for 4 h. The supernatant was discarded by centrifugation at 8000 rpm for 10 min. The precipitate was suspended with 20 mM Tris–HCl (pH 8.0) followed by sonication on the condition of 5s-on, 7s-off for 10 min. After that, the protein samples from both the supernatant and the precipitant were collected again by centrifugation at 8000 rpm for 10 min at 4°C and analyzed by sodium dodecyl sulfate polyacrylamide gel electrophoresis (SDS-PAGE).

### Western Blot

After the same amount of protein samples were separated by SDS-PAGE, the unstained gels were electro-transferred onto immune-blot polyvinylidene difluoride (PVDF) membranes (Bio-Rad, Hercules, CA, United States). Following blocked with 5% skim milk in PBST buffer for 1 h, an anti-his antibody (TransGen Biotech) was added at a 1:2000 dilution and incubated overnight. After washed three times with PBST buffer, the PVDF membrane was treated with goat anti-mouse IgG alkaline phosphatase-conjugated secondary antibody at a dilution of 1:1000 (Bio-Rad) for 2 h and washed again. The position of the bound antibodies on the membrane was detected by submerging it in a solution containing nitroblue tetrazolium (NBT) and 5-bromo-4-chloro-3-indolyl phosphate (BCIP), following a standard protocol.

### β-Galactosidase Assay

Plasmid pIs284 that contained a *lac*Z reporter gene was used for assaying the transcriptional levels. A series of nested fragments from *yneP* promoter region were fused to a promoter-less *lacZ* gene in pIS284. The truncated promoter regions were digested with *Hind*III and *Eco*RI, and then inserted into the similarly digested pIs284 plasmid. Finally, the successfully constructed plasmids, including BSP1, BSP2, BSP3, BSP4, BSP5, BSP6, BNP1, BNP2, BNP3, BNP4, BNP5, and BNP6, were transformed into *B. subtilis* 168 or *B. nematocida* B16, respectively. After the bacterial strains were cultured in LB medium for 30 h, β-galactosidase activities were determined with GENMED-galactosidase assay kit.

### SPME-GC-MS Analysis

A headspace solid-phase microextraction (SPME) method in combination with GC-MS was employed to detect methylketones, including 2-heptanone, in different bacterial cultures. The methods of sample preparation and detection were similar to our previous reports (Niu et al., 2010). Briefly, bacterial cultures were placed in 15 ml vials with a magnetic stirrer and extracted with the extraction head (CAD-PDMS 75 μm, Supelco). The fiber was exposed to the headspace above the sample at 65°C for 60 min followed by inserting into the GC injector of Clarus 500 GC-MS System (PE Co., Waltham, MA, United States) and desorbing at 250°C for 2 min. DM-5 column (DM-5 30 m × 0.32 mm × 1 μm) (Chrompack, Mid-delburg, Netherlands) was used in our GC-MS analysis. The initial oven temperature was 50°C, held for 2 min, ramped 4°C/min to 180°C and then 6°C/min to 280°C, and held for 10 min. The volatile components were detected by mass spectrometry with electron impact ionization at 70 eV, with a continuous scan for mass to charge ratio (m/z) from 35 to 550. Compounds were identified by matching the mass spectra with standards in the NBS 2005 library and Nist 2005 library.

The bacterial strains for SPME-GC-MS assay include *B. nematocida* B16, *B. subtilis* 168, *E. coli* BL21 with heterologously expressed YneP and YtpA, and *B. subtilis 168* overexpressing YneP’.

### Real-Time PCR Assays

The total RNA was isolated using an RNA extracting kit (Tiangen, China) following the treatment of DNaseI to avoid DNA contamination. RNAclean Kit (BioTeck, China) was then employed to further purify the total RNA. RNA concentration was determined by measuring absorbance at 260 nm using a UV spectrophotometer. After random-primed cDNAs were generated, qPCR analysis was performed with SYBR Green JumpStart Taq Ready Mix for qPCR kit (Sigma-Aldrich Co.) following manufacturer’s instructions. The partial sequence of 16S rRNA was used as an internal control. The PCR amplification used 40 cycles of 94°C for 30 s, 60°C for 30 s, 72°C for 40 s on ABI PRISM 7000 Real-Time PCR. The real-time PCR experiments were repeated three times for each reaction using independent RNA sample.

### Bioassay for Nematocidal Activity

The test bacterial strains were grown overnight at 37°C with 200 rpm shaking in 3 ml YPD (Yeast Extract/Peptone/Dextrose) medium. Then each 100 μl bacterial culture was diluted with M9 medium to 600 μl and transferred into 16-well plates. Seeded with L4 stage hermaphrodite nematodes (30–60 per well), the infection assay was performed at 25°C for different time points. Mortalities of nematodes were defined as the ratio of dead nematodes over the total number of tested nematodes. All infection experiments were performed in triplicates and were repeated at least three times.

### Chemotaxis Assays

Chemotaxis assay was performed based on the modified method reported previously (Stein and Murphy, 2014). In the chemotaxis assays, we used 10 cm-diameter tissue culture Petri dishes containing 10 ml of 1.6% agar each. Two marks were made on the back of the plate at opposite sides of the plate about 0.5 cm from the edge of the agar. The tested bacteria and the control (*E. coli* P50) were at the attractant source and the counter-attractant source, respectively. At the same time, 1 μl sodium azide with the concentration of 1 M was placed to anesthetize animals within about a 0.5 cm radius of the attractant or counter-attractant. After the tested worms of *C. elegans* were placed near the plate center, the numbers of animals at the attractant and the counter-attractant areas were determined within 1 h. The chemotaxis index was calculated as (animal numbers at attractant - animal numbers at counter-attractant)/total animal number.

### Statistical Analysis

All the data were expressed as the mean ± standard deviation (SD). Statistical comparisons were performed by a one-way analysis of variance (ANOVA) followed by Dunnet-*t* test.

## Results

### A Novel Methylketone Synthetase *YneP* in *B. nematocida* B16

**FIGURE 1:**
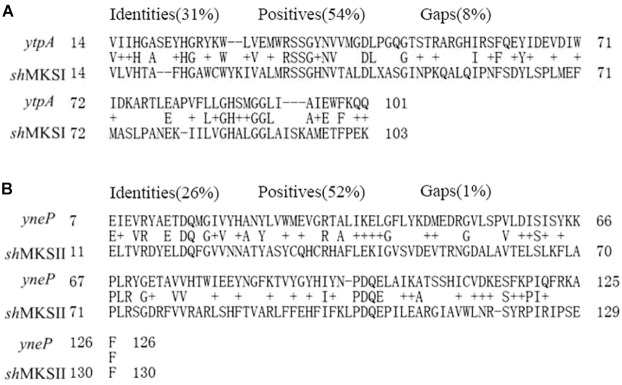
Sequence alignment of the *YtpA* gene to *sh*MKSI from *Lycopersicon hirsutum* f. *glabratum* **(A)** and *YneP* to *sh*MKSII **(B)**.

To investigate that the potential methylketone synthetase existed in our bacterial strain, we aligned *sh*MKSI (AY701574) and *sh*MKSII (EU883793) from wild tomatoes with the annotated genome of *B. nematocida* B16 at amino acid level, and two genes of *ytpA* and *yneP* were identified as having the highest sequence identity to *sh*MKSI and *sh*MKSII. The sequences of *ytpA* and *yneP* were then submitted in GenBank with accession number of MH687930 and MH687929, respectively. Compared to the 265-amino acid *sh*MKSI and the 149-amino acid *sh*MKSII, the full ORF of *ytpA* was 777 bp and encoded 259 amino acids, which was annotated as a putative lysophospholipase. The other gene *yneP* was 414 bp long and encoded a 138-amino acid hypothetical protein with unknown function. Between them, YtpA shared 31% identity and 54% positive amino acid sequence with *sh*MKSI (Figure 1A), and YneP shared 26% identity and 52% positive with *sh*MKSII (Figure 1B).

**FIGURE 2:**
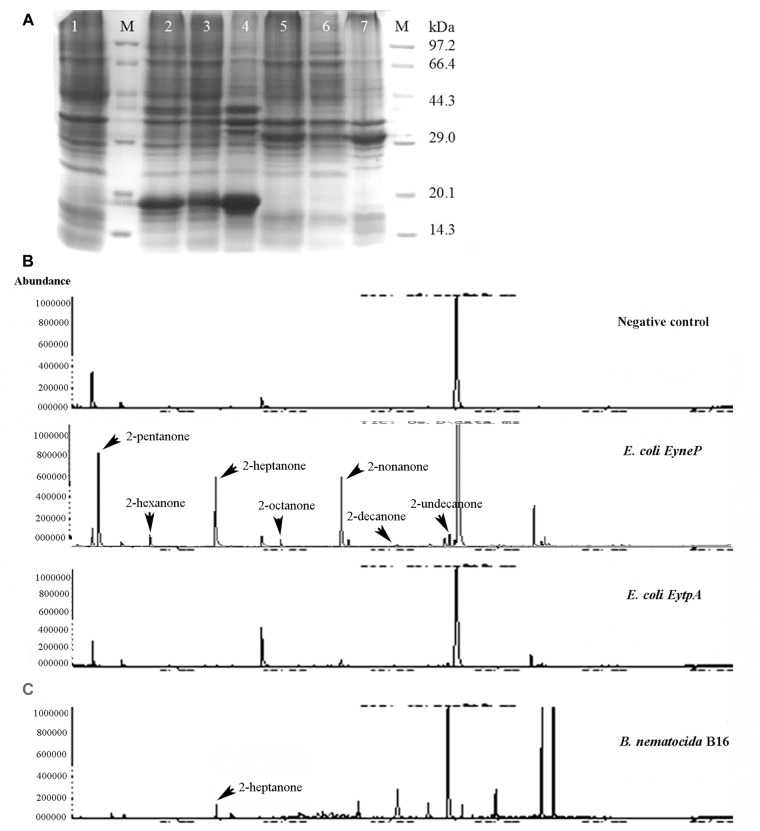
YneP heterologously expressed in *E. coli* synthesizes a variety of methylketone with medium chain length and functions as a methylketone synthase. **(A)** SDS-PAGE confirmed expression of the recombinant proteins of YtpA and YneP in *E. coli* BL21. Lane M represented the protein Marker; Lane 1–7 showed the negative control of *E. coli* BL21 with blank vector pET28a, *E. coli* BL21 overexpressing *yneP*, the supernatant of *E. coli* BL21 containing *yneP*, the precipitate of *E. coli* BL21 containing *yneP*, *E. coli* BL21 overexpressing *ytpA*, the supernatant of *E. coli* BL21 containing *ytpA*, the precipitate of *E. coli* BL21 containing *ytpA*, respectively. Arrowhead and arrow represented the proteins of YneP and YtpA expressed in *E. coli* BL21, respectively. **(B)** GC-MS analyses of the VOCs produced by the negative control *E. coli* BL21 with blank vector, *E. coli* BL21 with YtpA overexpression and *E. coli* BL21 with YneP overexpression. **(C)** GC-MS analyses of the VOCs produced by *B. nematocida* B16 strain.

In an attempt to examine whether *ytpA* and *yneP* function as methylketone synthase, we tried to construct *ytpA* or *yneP* gene knockout strain in *B. nematocida* B16. Though the pCP115-*yneP* and pCP115-*ytpA* recombinant plasmids for knocking-out *ytpA* and *yneP* genes were successfully constructed, the positive transformants of *B. nematocida* B16 were never obtained (data not shown). Thus, heterologous expressions of those two genes were further carried out in *E. coli* BL21, and the changes of methylketone production were determined using SPME-GC-MS analysis. First, the SDS-PAGE analysis confirmed that the recombinant proteins of YtpA and YneP were expressed and had the molecular weight of 29.5 and 16.1 kDa as expected (Figure 2A). Then, we compared the VOCs produced by the *E. coli* BL21 with a blank vector, *E. coli* BL21 with *ytpA* and *E. coli* BL21 with *yneP*. The results from SPME-GC-MS revealed that 2-pentanone, 2-hexanone, 2-heptanone, 2-octanone, 2-nonanone, 2-decanone, 2-undecanone, 2-tridecanone were detected in the VOCs of *E. coli* BL21 when YneP was overexpressed (Figure 2B). The peak area of methylketones with odd-numbered carbon were more prominent, such as 2-pentanone, 2-heptanone, and 2-nonanone with peak areas reaching 6.66, 6.21, and 4.8%, respectively (Table 3). Furthermore, the peak area of 2-heptanone far exceeded the 0.16% in the positive control of *B. nematocida* B16 (Figure 2C). In contrast, methylketones were undetectable under the same condition in *E. coli* BL21 with YtpA overexpression, which was similar to the result from the negative control of wild type *E. coli* BL21 (Figure 2B). Altogether, our current results suggest that *yneP*, but not *ytpA*, functions as the methylketone synthase.

**Table 3:** SPME-GC-MS analyzed the production of methylketones in *E. coli* BL21 heterologously expressing *yneP*.

RT	Peak area (%)	Compounds	CAS	Quality
1.423	6.66	2-Pentanone (C5)	000107-87-9	91
3.805	1.08	2-Hexanone (C6)	000591-78-6	91
6.828	6.21	2-Heptanone (C7)	000110-43-0	91
9.840	0.49	2-Octanone (C8)	000111-13-7	93
12.646	4.80	2-Nonanon (C9)	000821-55-6	97
15.234	0.14	2-Decanone (C10)	000693-54-9	92
17.651	0.73	2-Undecanone (C11)	000112-12-9	94
22.051	0.62	2-Tridecanone (C13)	000593-08-8	93

*RT, retention time; CAS, chemical abstract service number; Quality, credibility score.*

### Enhancement of *de novo* Fatty Acid Synthesis During 2-Heptanone Production

**FIGURE 3:**
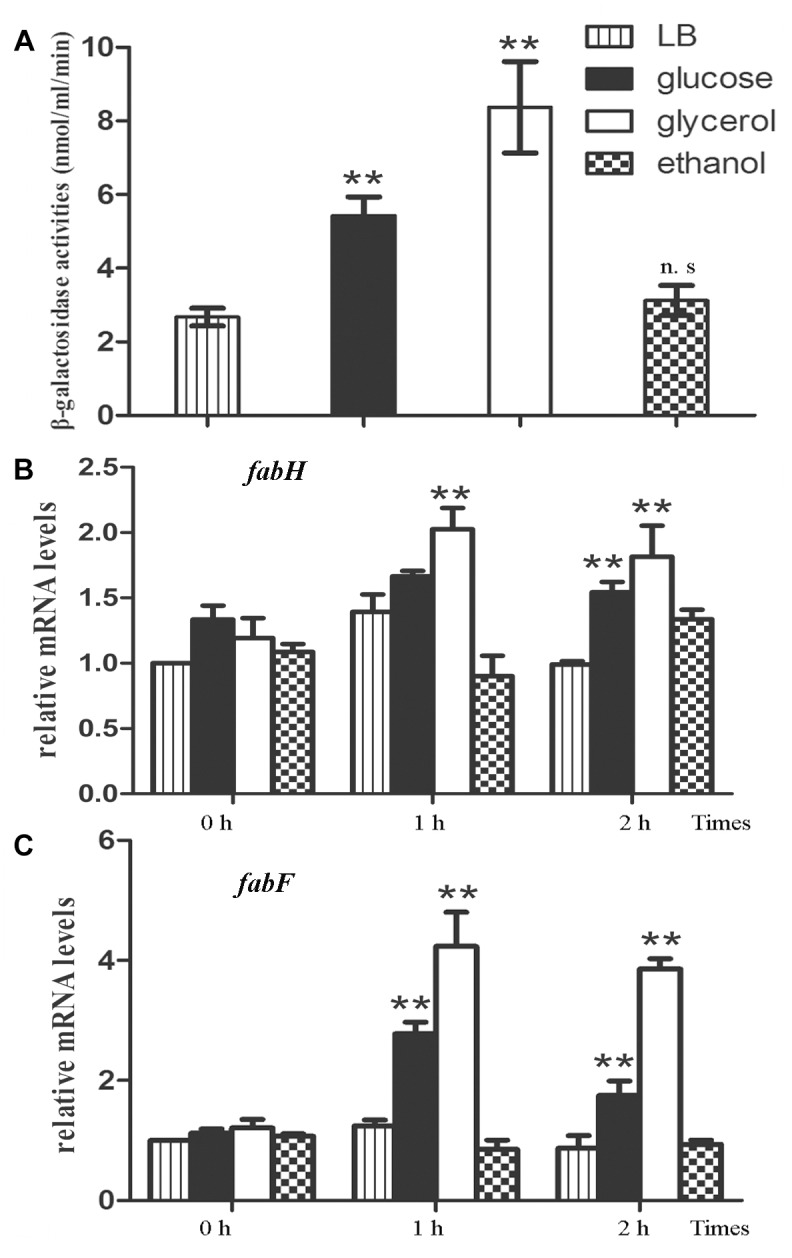
The methylketones in *B. nematocida* B16 are synthesized during *de novo* fatty acid synthesis. **(A)** The transcriptional levels of *yneP* gene were determined by assaying β-galactosidase activities when induced by the different carbon sources. **(B,C)** qPCR was used to determine the relative mRNA levels of *fabH* and *fabF*, the key enzymes in *de novo* fatty acid synthesis. ^∗∗^*P* < 0.05.

Based on the fact that the methylketone synthetases *sh*MKSI and *sh*MKSII in wild tomatoes catalyze the substrate β-ketoacyl-ACP (β-ketoacyl-acyl carrier protein) which is one of the intermediate products in fatty acid synthesis (Fridman et al., 2005), it is reasonable to speculate that the methylketones in *B. nematocida* B16 are also synthesized via the *de novo* fatty acid synthesis pathway rather than the abortive degradation pathway. To test this hypothesis, we fused the promoter region of *yneP* to the reporter plasmid pIs284 to drive the expression of β-galactosidase. We determined the transcriptional levels of *yneP* by assaying β-galactosidase activities when induced by the different carbon sources including glucose, ethanol, and glycerol. Our results showed that, compared to the control in LB medium, the activities of β-galactosidase ranged from high to low levels in the following order: glycerol, glucose, and ethanol (Figure 3A). Considering that glycerol is closer than glucose to pyruvic acid in the glycolysis pathway, our result implied that the methylketones in *B. nematocida* B16 were likely synthesized through the *de novo* fatty acid synthesis pathway.

To further validate our hypothesis, the transcriptional levels of β-ketoacyl-ACP synthase II (*fabF*) and β-ketoacyl-ACP synthase III (*fabH*), two key enzymes in the *de novo* fatty acid synthesis, were determined again under the above carbon sources using qPCR. Between them, FabH catalyzes the quintessential ketoacyl synthase reaction with malonyl ACP and acetyl CoA, and FabF catalyzes the related reaction. As expected, the relative mRNA levels of *fabH* and *fabF* in glycerol-contained medium were the highest among the three media (followed by glucose and ethanol) and increased by about two or fourfolds within 2 h, respectively (Figure 3B,C).

### Absence of 2-Heptanone in VOCs From the Non-pathogenic *B. subtilis* 168

**FIGURE 4:**
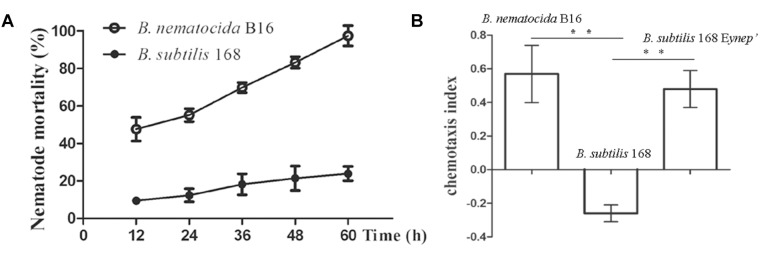
The non-pathogenic bacterium *B. subtilis* 168 shows little ability to kill or attract nematodes. **(A)** The nematotoxic activities of *B. nematocida* B16 and *B. subtilis* 168. **(B)** The attractive capabilities for *C. elegans* in the different bacterial strains of *B. nematocida* B16, *B. subtilis* 168, and *B. subtilis* 168 homolously expressing *yneP’*. ^∗∗^*P* < 0.05.

**FIGURE 5:**
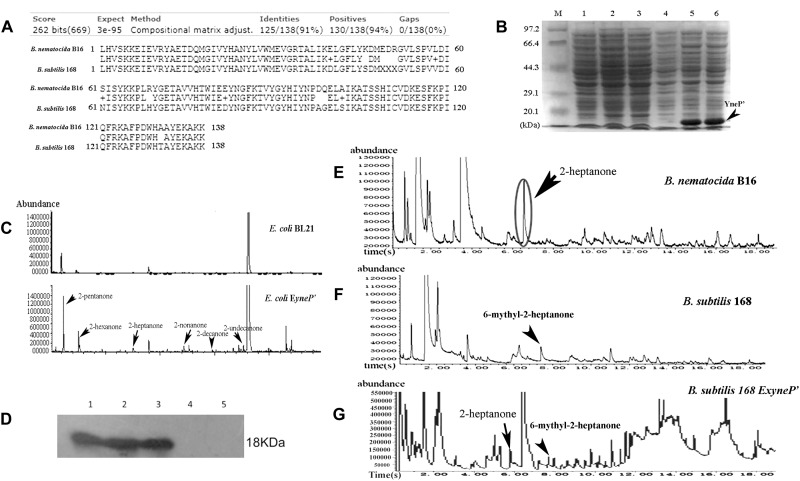
The *yneP’* gene in the non-pathogenic *B. subtilis* 168 has the methylketone synthetase activity but absence of 2-heptanone in the VOCs from *B. subtilis* 168. **(A)** The YneP’ in *B. subtilis* 168 showed highly homologous to YneP in *B. nematocida* B16. **(B)** SDS-PAGE confirmed expression of the recombinant protein of YneP’ in *E. coli* BL21. Lane M, the protein Marker; lane 1 and 2, the negative control of *E. coli* BL21 with blank vector; lane 3 and 4, the strain *E. coli* BL21 with Ex*yneP’* without IPTG induction; lane 5 and 6, the heterologous expression of YneP’ in *E. coli* BL21 after induced with 0.1 mM IPTG. The molecular size of target protein is about 18 kDa. **(C)** GC-MS analyses of the VOCs from *E.coli* Ex*yneP’* and the negative control. **(D)** Western blot to the homolously expressed YneP’ with 6 × his tag using anti-his antibody. Lane 1–3 represented the three positive transformants of *B. subtilis* 168 Ex*yneP’*; Lane 4 and 5 represented the wild type *B. subtilis* 168 and *B. subtilis* 168 with the blank vector pDG148. **(E–G)** Comparisons of the VOCs among *B. nematocida* B16, *B. subtilis* 168, and *B. subtilis* Ex*yneP’.* Among those three strains, the peak of 2-heptanone was detected in *B. nematocida* B16 but absence in *B. subtilis* 168. Instead, a peak of 6-methyl-2-heptanone could be detected in *B. subtilis* 168. But 2-heptanone in VOCs reappeared when overexpressing *yneP’* in *B. subtilis* 168.

The saprobiont bacterium *B. subtilis* 168 was one of the closest relatives of *B. nematocida* in the *Bacillus* genus based on the 16s rDNA phylogenetic tree, but it obviously had little nematocidal activity compared to the strain of *B. nematocida* (Figure 4A) (Deng et al., 2013). Therefore, we tested whether *B. subtilis* 168 could attract nematodes or produce the attractant 2-heptanone. In chemotaxis assays, the worms immediately oriented their movements and then they congregated toward *B. nematocida* B16. The chemotaxis index of *B. nematocida* B16 was about 0.57 ± 0.17, which meant that our strain showed a stronger attractive capability for nematodes compared to the counter-attractant *E. coli* OP50 (Figure 4B). Contrarily, the bacterium *B. subtilis* 168 showed little ability to lure nematodes with the chemotaxis index of −0.26 ± 0.05 (Figure 4B). The VOCs produced by *B. nematocida* B16 and *B. subtilis* 168 were further assayed. Our results demonstrated the presence of 2-heptanone in *B. nematocida* B16 (Figure 5E), but it was not detected in *B. subtilis* 168 (Figure 5F).

### A Homology *yneP’* With Methylketone Synthetase Exists in *B. subtilis* 168

Since neither attractive capability nor 2-heptanone was found in *B. subtitis* 168, we speculated that the homolog of *yneP* either does not exist or if it exists, it could be a pseudogene. To address this issue, we aligned *yneP* of *B. nematocida* B16 with the genome of *B. subtilis* 168 and found that YneP’ in *B. subtilis* 168 shared 91% identity and 94% positive amino acid sequence with that of *B. nematocida* B16 (Figure 5A). To determine whether *B. subtilis* 168 *yneP’* had the same synthase activity, the ORF of *yneP’* was amplified, cloned into pET28a, and expressed in *E. coli* BL21 with a molecular weight as we had expected (Figure 5B). Next, we verified its activity as a methylketone synthase by GC-MS. Compared with the negative control of *E. coli* BL21, a variety of methylketons were identified, which meant that the heterologously expressed YneP’ of *B. subtilis* 168 indeed functioned in methylketone biosynthesis (Figure 5C).

To further confirm its activity *in vivo*, a homologous expression of YneP’ fused with 6 × his tag (*B. subtilis* 168 *EyneP’*) was achieved in *B. subtilis* 168. Production of the target protein was validated by Western blot using anti-his antibody (Figure 5D). Measuring the VOCs produced by *B. subtilis* 168 *EyneP’* using GC-MS, the peak of 2-heptanone became more obvious compared to the negative control (Figure 5G). Furthermore, in chemotaxis assay, *B. subtilis* 168 overexpressing *yneP’* showed a stronger attractive capability for nematodes (Figure 4B), though the chemotaxis index was still lower than *B. nematocida* B16.

### Metabolic Transformation but Not Transcriptional Level Responsible for the Difference of 2-Heptanone in the Two Bacteria

**FIGURE 6:**
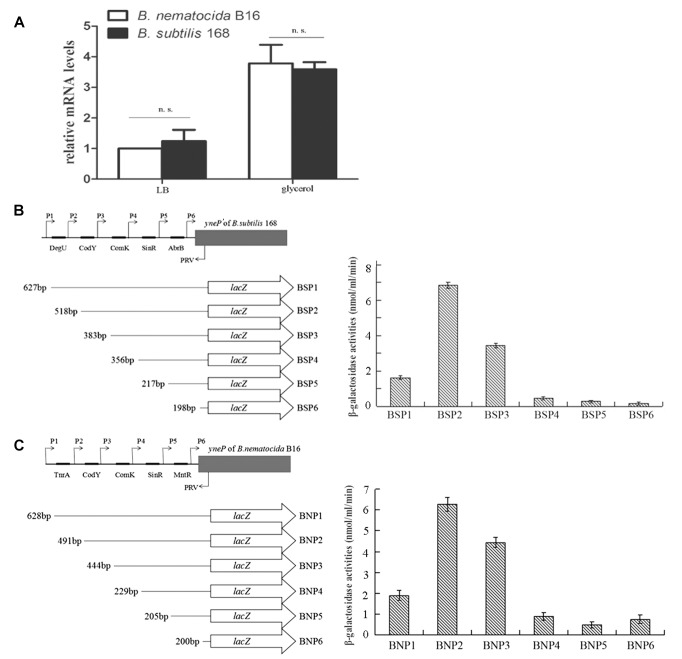
The transcriptional levels of *yneP* of *B. nematocida* B16 and *yneP’* of *B. subtilis* 168. **(A)** qPCR detected the relative mRNA levels of *yneP* of *B. nematocida* B16 and *yneP’* of *B. subtilis* 168 under the same conditions. n.s., *P* ≥ 0.05. **(B)** Assays to β-galactosidase activities in the series of *yneP’*::lacZ fusions. Schematic representation of the different *yneP’::lacZ* fusions used in this study. The filled arrows indicate the primers used for generating the various reporter fusions (P1–PRV), whereas the 5’ and 3’ end termini of the *yneP’::lacZ* fusions are denoted with their nucleotide positions relative to the initiator codon. The black oblongs indicate the binding motifs in the promoter of *yneP’* of *B. subtilis* 168. **(C)** Assays to β-galactosidase activities in the series of *yneP*::lacZ fusions of *B. nematocida* B16.

Based on our experimental evidence that the gene *yneP’* of *B. subtilis* 168 had the activity of methylketone synthase, it was reasonable to speculate that the absence of 2-heptanone in this strain was likely due to the repressed expression of *yneP’*. To test this assumption, we firstly compared the mRNA levels of the methylketone synthase gene between *B. nematocida* B16 and *B. subtilis* 168 by qPCR. Our results showed the similar transcriptional levels in those two strains under the same conditions (Figure 6A).

The *cis*-acting elements within those two promoters were further analyzed by the reporter gene *lacZ*. Using the software DBTBS, the predicted transcription factor binding sites of DegU, CodY, ComK, SinR, and AbrB were found in the promoter region of *B. subtilis* 168 *yneP’*; while TnrA, CodY, ComK, SinR, and MntR were found within the promoter of *B. nematocida* B16 *yneP* (Figure 6B,C). After a series of reporter fusions containing the truncated promoter regions to *lacZ* were constructed (BSP1, BSP2, BSP3, BSP4, BSP5, BSP6 of *B. subtilis* 168 and BNP1, BNP2, BNP3, BNP4, BNP5, BNP6 of *B. nematocida* B16, respectively) and transformed into their host bacterial strains, β-galactosidase activity of each strain was measured when cultured in LB medium for 30 h. Our data showed that most of the *cis*-acting elements analyzed in *B. subtilis* 168 positively regulated the expression of *yneP*’ except for DegU (Figure 6B). To the *yneP* promoter of *B. nematocida* B16, TnrA and MntR negatively regulated *yneP* expression, but CodY, ComK, and SinR had positive effects (Figure 6C). Furthermore, the quantifications of β-galactosidase activities were similar between those two bacterial strains. Therefore, our results indicate that, under the same tested conditions, the transcription control of *yneP’* in *B. subtilis* 168 was not more strongly suppressed than *B. nematocida* B16.

To further understand the reason for the lack of 2-heptanone in *B. subtilis* 168, we scanned each peak of GC-MS again and found a peak corresponding to 6-methyl-2-heptanone detected in both *B. subtilis* 168 and *B. subtilis* 168 E*yneP’* (Figure 5F,G), but no appearance in the VOCs of *B. nematocida* B16. Our results suggest that 2-heptanone synthesized by the methylketone synthase YneP’ could be methylated and transformed into 6-methyl-2-heptanone upon its production in *B. subtilis* 168.

## Discussion

The prevalence of methylketones with an odd number of carbons in nature suggests that they are most likely derived from decarboxylation of the corresponding β-ketoacids that are intermediates in either the biosynthesis or the degradation pathways of fatty acids. Of the two pathways, the spontaneous decarboxylation is more rationally expected since CoA esters and free β-ketoacids, the intermediates in fatty acid degradation are unstable. Wild tomatoes produce methylketones as repellants against its pests (Yu, 2013), in which two genes *shMKSI* and *shMKSII* are identified to be responsible for the production of methylketones during fatty acid biosynthesis (Yu et al., 2010). In this pathway, ShMKS2 hydrolyzes β-ketoacyl-A to release 3-ketoacids, and then ShMKS1 catalyzes the decarboxylation of 3-ketoacids to produce methylketones (Yu et al., 2010; Yu and Pichersky, 2014). So the *shMKSI* gene is epistatic to *shMKSII* in the pathway for methylketone biosynthesis. MKS homologs have also been found in other plants and non-plant organisms (Nikolau et al., 2008). However, there has been limited experimental evidence supporting these MKS homologs really have the synthetase activities of methylketones with different chain length. In the genome of *B. nematocida* B16, *ytpA* and *yneP* were identified as the homolog of *shMKSI* and *shMKSII*, respectively. The *yneP* gene was predicted as an unknown thioesterase, which belonged to a large group of hydrolytic enzyme superfamily with a “hot-dog”-like structure. Its activity as a methylketone synthase was verified when the methylketones with medium chain length were obtained in *yneP*-overexpressed *E. coli* but absence in the negative control. Furthermore, the data from transcriptional analyses also supported that the methylketones in *B. nematocida* B16 were synthesized during *de novo* fatty acid synthesis rather than via the degradation pathway. The other *YtpA* gene, though homologous with the tomato *shMKSI*, showed no obvious contribution to methylketone production. Therefore, in *Bacillus* sp., we hypothesize that *yneP* likely catalyzes β-ketoacyl-A, the intermediates of fat acid synthesis, into 3-ketoacids which are then further decarboxylated spontaneously. But the role of YtpA, the homolog of *shMKSII* in *B. nematocida* B16, remains to be confirmed.

In nature, 2-heptanone functions as an important allomone in the interaction between prey and predator. For example, when worker bees attack their enemies by sting, they often use the upper jaw to bite the enemy. Synchronously, the chemicals, such as 2-heptanone, are left on the enemy’s bodies to guide the other bees for attacking (Schearer and Boch, 1965). Furthermore, one yeast, *Kodamaea ohmeri*, carried by honeybees can produce more abundant 2-heptanone using the pollen collected in honeycomb, which attracts members of the intruder *Aethina tumida* and leads to disastrous damages for honeybees (Torto et al., 2007; Benda et al., 2015). Interestingly, our previous investigations have also illustrated that 2-heptanone produced by the bacterial pathogen *B. nematocida* B16 strain can successfully lure the worms and fulfill one of the prerequisites for a Trojan horse-like infection (Niu et al., 2010). But the evolutionarily closely related strain *B. subtilis* 168 showed little attraction for nematodes, even though the gene *yneP*’ had methylketone synthetase activity. The similar expressional levels of the homologs between those two bacteria form stark contrast to their vast differences in both 2-heptanone production and attractive capability. Our data further suggest that instead of producing 2-heptanone as a final product, 2-heptanone was likely converted into 6-methyl-2-heptanone in *B. subtilis* 168. This metabolic transformation may be an effective strategy to prevent the non-pathogenic *B. subtilis* 168 from being the food of nematodes. Our results provided a co-evolution paradigm of second metabolites during the interactions between pathogenic/non-pathogenic bacteria and host.

## Author Contributions

XH and KZ designed the research. MZ, XX, YL, and PW carried out the experiments. KZ and SN analyzed the data. XH and MZ wrote the manuscript.

## Conflict of Interest Statement

The authors declare that the research was conducted in the absence of any commercial or financial relationships that could be construed as a potential conflict of interest.
